# Vaccine trials during a pandemic: potential approaches to ethical dilemmas

**DOI:** 10.1186/s13063-021-05597-8

**Published:** 2021-09-15

**Authors:** Manaf Alqahtani, Saad I. Mallah, Nigel Stevenson, Sally Doherty

**Affiliations:** 1 The National Taskforce for the Combating of the Coronavirus (COVID-19), Manama, Kingdom of Bahrain; 2 The National COVID-19 Research and Ethics Committee, Manama, Kingdom of Bahrain; 3 Department of Medicine, Royal College of Surgeons in Ireland – Bahrain, Busaiteen, Kingdom of Bahrain; 4 School of Medicine, Royal College of Surgeons in Ireland – Bahrain, Busaiteen, Kingdom of Bahrain; 5 Department of Biochemistry and Immunology, Trinity Biomedical Sciences Institute, Trinity College Dublin, Dublin, Ireland; 6 Research Ethics Committee, Royal College of Surgeons in Ireland – Bahrain, Busaiteen, Kingdom of Bahrain

**Keywords:** COVID-19, Medical ethics, Pandemic, Vaccines, Clinical trials

## Abstract

Ever since the emergence of the coronavirus disease 2019 (COVID-19), global public health infrastructures and systems, along with community-wide collaboration and service, have risen to an unprecedented challenge. Vaccine development was immediately propelled to the centre of all our scientific, public health and community efforts. Despite the development of SARS-CoV-2 vaccines arguably being the greatest and most palpable achievements of the past 12 months, they have also been one of the most contentious and debated issues during the pandemic. However, what uniquely differentiates vaccine development is its intimate relationship with the community it seeks to serve; both in its clinical trial testing as an efficacious and safe prophylactic, and its post-developmental ‘roll-out’ success, as an effective public health tool. These relationships have birthed a myriad of complexities, from community-based mistrust, to academically contended ethical dilemmas. Indeed, the accelerated advances in the COVID-19 vaccine race have further exacerbated this phenomenon, bringing with it new ethical dilemmas that need to be examined to ensure the continued clinical success of these therapeutics and a renewed societal trust in clinical medicine.

In this paper, we discuss two major ethical dilemmas: (1) the equipoise of continuing new vaccine trials in the advent of successful candidates and (2) the maleficence of blinded placebo arms. Accordingly, we discuss six different potential approaches to these ethical dilemmas: (1) continuing with placebo-controlled trials, (2) transitioning from placebo-controlled to open-label, (3) unblinding at-risk priority groups only, (4) transitioning to a blinded stepped-wedge cross-over design, (5) progressing to a blinded active-controlled stepped-wedge cross-over trial, and (6) conducting randomised stepped-wedge community trials. We also propose a decision-making algorithm for relevant stakeholders in advanced stages of vaccine trials.

It is important to remember that the emergent nature of the COVID-19 situation does not justify a compromise on core ethical values. In fact, the discourse surrounding this topic and the decisions made will remain a potent case study and a continuously referenced example for all such future scenarios.

## Introduction

With the start of the COVID-19 pandemic, several institutions from around the world immediately announced their participation in the development of an effective and safe vaccine against SARS-CoV-2. As of today, July 2021, marking over a year and a half since the beginning of the outbreak, there has been over 100 vaccines in development [1]. Eight vaccines—including most notably Pfizer-BioNTech, Moderna, Oxford-AstraZeneca and Sinopharm—have received full approval for use by various countries, while eight others have received limited or emergency use authorization. Overall, there are currently 32 vaccines in phase III testing, 37 in phase II, and 50 in phase I [1].

In our long human history with infectious diseases, this is the first time that effective vaccines are developed and made available in the midst of a novel ongoing pandemic [2], bringing along an unprecedented challenge. Nonetheless, global discourse on alternative trial designs to the standard placebo-controlled randomised controlled trials (RCTs) may not be unique to the current pandemic and can be dated back to the Ebola and Zika virus outbreaks [3]. At first glance, it may appear that the larger the number of vaccines in development, the more likely that a more efficacious one is discovered, and that a longer clinical trial follow-up period would yield more informative data and a safer vaccine. However, while the unprecedented global pressure of the COVID-19 pandemic has undoubtably enhanced scientific advancement, the continual rise in infections and deaths, along with the emergence of new, more infectious strains of the virus [4], adds a heavily weighted sense of urgency that must not be allowed to distort scientific and clinical integrity. Indeed, while encouraging scientific discoveries, these continual pressures should also bring medical ethics to the forefront of discussions, to ensure that only effective and safe vaccines, that were tested in ethically-sound trials, see the light of clinical use.

In order to best evaluate the efficacy and safety profile of a new vaccine candidate, randomised, double-blinded, placebo-controlled trials are conducted [5]. The phase III COVID-19 vaccine trials thus often include a large group of participants, half of whom receive a placebo while the other half receives the experimental vaccine. Both groups are monitored for several months and surveyed for side-effects and incidence of newly-acquired COVID-19 infection. For the clinical and ethical safeguarding and success of human clinical trials, the principle of ‘equipoise’ must always be at the forefront of consideration [6, 7]. In other words, there must exist genuine uncertainty in the scientific and medical community regarding the comparative therapeutic benefits of these vaccines throughout their development, subsequent licensing, and when assessing their ‘real-life’ effectiveness. It is worth noting that alternative trial designs that may, in certain contexts, be more advantageous than placebo-controlled RCTs have received attention in light of the viral outbreaks witnessed in the last few years [3]. As per the Global Forum for Bioethics in Research, alternative approaches to trial design must consider their scientific validity, ethicality and practicality [3]. While continually using the available data to make an informed choice, without a measured ethical approach, we risk the health and wellbeing of the global population and public trust for future generations of therapeutic development. This may be of even greater significance in the current context and any such similar instances moving forward, where enrolment and retainment of participants may be at risk of insufficiency due to the accelerated number of trials and thus demand for participants, as well as the heightened uncertainty factor and complex negative societal perspectives of COVID-19 [8, 9]. Here, we propose two major ethical issues for debate and propose potential approaches for immediate consideration, in what is likely to be the most important year of vaccine development and clinical care in our modern age.

## Dilemma 1: The equipoise of new vaccine trials in the advent of successful candidates

Whether the state of equipoise that was present at the start of the COVID-19 pandemic and its vaccine development still holds true today is questionable. Initially, a clear state of equipoise existed regarding the effectiveness of newly developed vaccines in both preventing severe COVID-19 disease and suppressing its spread. As discussed, earlier the concept of equipoise—that genuine certainty exists within the medical community regarding the superiority of one intervention over another—is necessary for the safeguarding of trial participants and research integrity. Furthermore, Freedman et al. have highlighted that a state of equipoise is not only necessary for the initiation of a trial, but rather for its continuation as well; thus, if equipoise is disturbed during the course of a trial, it is expected that it would be terminated, and all participants offered the superior intervention [6].

With the release of phase III clinical trial results from Pfizer-BioNTech [10], Moderna [11] and Sinopharm [12], currently approved vaccines are evidently, and without clinical or statistical uncertainty, more efficacious (72–95%), than placebo-controls in preventing COVID-19 disease or reducing its severity. As such, with such a range of successful preventative countermeasures now on offer, it could be argued that the initial state of equipoise that existed at beginning of the COVID-19 vaccine race can no longer be reasonably justified. Yet, there remains a large number of vaccine trials being conducted globally, which are enrolling thousands of individuals, many of whom are in ‘high risk’ groups (e.g. healthcare professionals, elderly) [1]. While these trials may generate new effective and safe candidates, the participants are—by default—not being provided with the provenly efficacious vaccines already available. As such, it could be argued that all individuals who are eligible to receive a proven efficacious vaccine, should be provided with it, and that placebo-controlled vaccine trials are no longer required nor ethically justified.

Alternatively, a state of equipoise may still be argued for. On the basis of maximal benefit, vaccines with improved immunogenicity/efficacy, fewer side effects/reactogenicity, easier transport, storage and administration and more favourable economic and logistical considerations—especially in the case of a global pandemic—may be worth the risks of placebo-controlled vaccine trials [7, 13]. Additionally, Miller et al., in reply to views previously presented by Freedman and colleagues, argue that the two main points against the ethicality of placebo-controlled trials in the presence of a provenly superior intervention—which include violating the physician’s therapeutic obligation to offer optimal care and the lack of scientific and clinical merit—erroneously conflate the ethics of clinical research with the ethics of clinical care. In reply to the first point, Miller et al. argue that the rationale and purpose behind placebo-controlled RCTs is to promote optimal scientific validity to answer clinical question with wide-extending repercussions, rather than optimal therapeutic benefit with repercussions personalised to an individual, as would normally be the case in clinical practice. Secondly, for the argument against scientific and clinical merit, Miller et al. invoke the methodological superiority of placebo-controlled trials—both in rigour, validity and efficiency—compared to active-controlled trials [14]. Nonetheless, as per the World Medical Association’s Declaration of Helsinki [15], the line is drawn when participants in the placebo-arm are subject to additional risks of ’serious or irreversible harm’ as a result of not receiving the standard intervention. What this translates to in the context of vaccine trials generally, and COVID-19 specifically, is unclear, and likely contentious.

It is important to consider that the political and international nature of COVID-19 vaccine development is likely to limit the discussions of equipoise and ethicality to a local as opposed to a trans-boundary global scale. Furthermore, while the vaccinations have been tested in heterogenous populations, their effects on different age groups and ethnicities are yet to be fully determined. This may encourage the continued exploration of new vaccine candidates.

Ideally, arguments in favour of the ethicality of placebo-controlled vaccine trials based on the logic of maximal benefit would be justified through a society-centred utilitarian lens, focusing on ‘the greater good for the greatest number’ [16]. On the other hand, as per a patient-centred deontological lens, such trials may not suffice the non-maleficence principle for the placebo-arm participants involved [17]. In modern medical ethics, and in accordance with the Declaration of Helsinki, the protection of the health and interests of study participants should always take priority [15]. This conflict is not restricted to new vaccine trials, but also forms a pertinent issue for the continuation of ongoing vaccine trials, which gives rise to the second ethical dilemma.

## Dilemma 2: The maleficence of blinded placebo arms

Vaccine trials, as per regulatory requirements, are designed to follow-up with participants for 1 to 2 years, which is the case with all three currently approved vaccine candidates [18–20]. This post-marketing surveillance allows for gathering of data on long-term efficacy and side effects [21]. As such, half of the participants of all current and future COVID-19 vaccine trials (which would cumulatively include hundreds of thousands of individuals), who are unknowingly in the placebo arm of the trial, would not receive the protective vaccine unless they drop out of the trial. Therefore, it is statistically inevitable that a significant number of these participants would eventually be infected with COVID-19, leading to their morbidity and/or mortality. Conversely, we can predict with a high degree of certainty that this illness and death could be avoided if participants are unblinded and offered the vaccine when phase III of clinical trials concludes. In line with the perspectives that were discussed in Dilemma 1, specifically those of the Declaration of Helsinki and Freedman et al., the ethicality of continuing with placebo-controlled trials may be argued against [6, 15]. Indeed, these issues raise major ethical dilemmas for all COVID-19 trials, as they are effectively withholding a proven disease countermeasure from the very individuals that risked their health and wellbeing to test its safety and efficacy for the greater good of society.

On the same note however, based on Miller et al.’s arguments, it may also be said that the continuation of placebo-controlled trials is necessary to preserve and uphold the integrity and validity of the data and is ethically sound as long as participants are not ‘exploited’. This is defined as not being exposed to ‘excessive risk’ and the participant understanding that they are participating in an experiment rather than receiving personalised medical care directed towards their best interest [14]. Furthermore, under the principle of autonomy, these participants were informed the potential risks and have therefore knowingly consented; as such, continuing with placebo-controlled trials may be justified, forming the basis of the first approach to this ethical paradox.

It is worth mentioning that an expert panel convened by the WHO Department of Ethics and Social Determinants in 2014 concluded that the risk-benefit profile of placebo-controlled vaccine trials may be acceptable when the following four criteria are met:

(1) The study question cannot be answered with an active-controlled trial design, (2) the risks of delaying or foregoing an existing efficacious vaccine are adequately minimised or mitigated, (3) the use of a placebo control is justified by the potential public health or social value of the research, and (4) the research is responsive to local health needs [22]. It is important no note however that these guidelines are concerned with the ethicality of such trials in a context different than the current one, where efficacious vaccines have become available during an active pandemic while large numbers of concurrent trials continue globally.

## Approach 1: Continuing with placebo-controlled trials

**Fig. 1:**
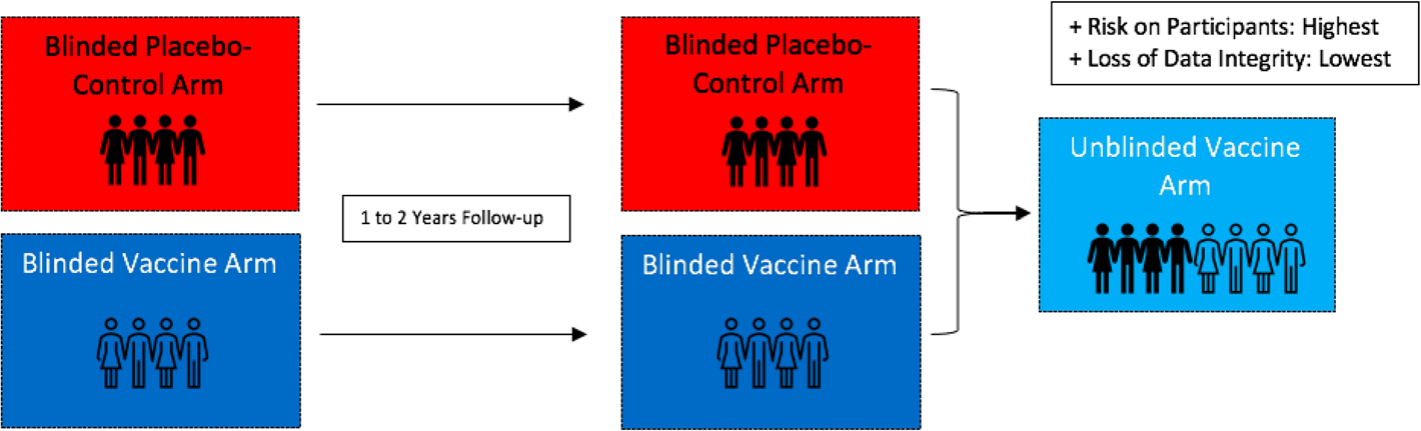
Continuing with placebo-controlled trials

Modern medical ethics for clinical research place the utmost importance and strictest of requirements for the obtainment of informed consent [15, 23]. As such, encouraging blinded placebo-controlled trials (Fig. 1) for both new and continuing vaccine trials for altruistic intents may be determined to be ethical, as long as all participants are properly informed and advised on the current global and local vaccine developments, the available options within and outside the trial, and the risks involved if they are in the placebo arm. Furthermore, considering that long-term side effects of COVID-19 vaccines are still unknown, a genuine uncertainty and risk, and thus a state of equipoise, may be argued for [5, 24]. As such, if the participant, judged to be capable of informed consent, decides to continue blinded to either arm, then the trial may be argued to be ethically sound. If such an option is opted for, we emphasise the importance of fully educating the participants of the information needed to provide an informed consent, using the most effective and appropriate mediums (e.g. creating informational and engaging videos as opposed to only sending a written leaflet).

Nonetheless, it should still be considered that as healthcare professionals and clinical researchers, a responsibility prevails to do the most good for our patients and study participants, and to limit and/or alleviate overt and implicit pressuring factors that may lead said individuals to make decisions that could risk their health and wellbeing. As such, transforming the trials to an open-label design may be a second approach.

## Approach 2: Transitioning from placebo-controlled to open-label

**Fig. 2:**
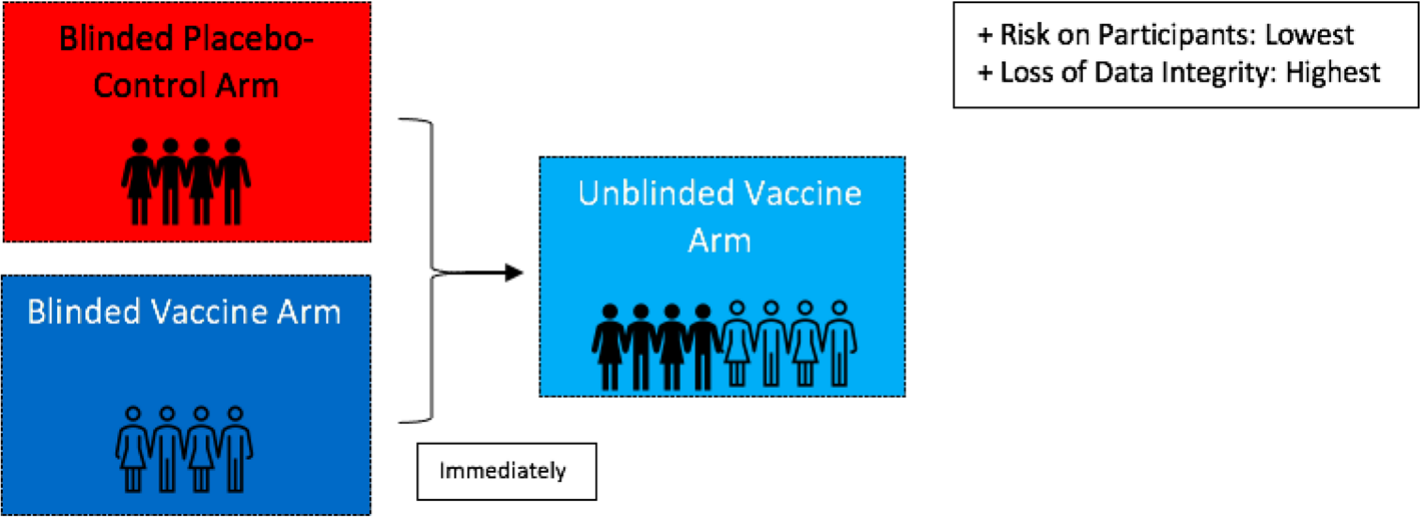
From placebo-controlled to open-label

An open-label approach, where all participants are unblinded and offered the vaccine, is currently being considered by manufacturers of approved vaccines (Fig. 2) [25]. This approach is supported first by the strong evidence for the efficaciousness of the vaccines over placebo and the overall positive safety profile. Second, this approach would prioritise study participants receiving the vaccine regardless of risk priority, which, considering that the risks they undertook were monumental for the success of the vaccine trials, may be justified by the principle of reciprocity [5]. This approach, however, may conflict with certain principles of health equity and justice, specifically in cases of limited vaccine supply [26]. As such, many individuals at high risk of infection and mortality from COVID-19 who did not enrol in a trial, such as healthcare professionals or the elderly, would consequently not receive a vaccination, in favour of healthy, young trial participants. Furthermore, by adopting this approach, beneficial follow-up data on long-term side-effects and duration of immunity would no longer be available [5]. As such, a third approach of only unblinding ‘at-risk’ participants may be considered.

## Approach 3: Unblinding at-risk priority groups only

**Fig. 3:**
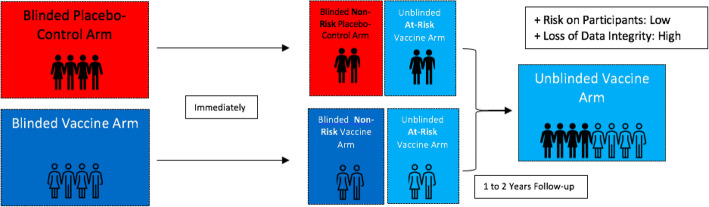
Unblinding at-risk priority groups

As a middle-ground, a risk-stratified unblinding approach which continues placebo-controlled trials for the majority but adopts an open-label approach for ‘at risk’ participants on basis of compassionate use may be more appropriate for the current global pandemic. This option would prioritise the unblinding and vaccination of trial participants who would normally be eligible for vaccination outside of the actual trial (Fig. 3). Two priority risk groups could be identified including (1) trial participants at higher risk of infection due to exposure (e.g. frontline personnel, healthcare professionals and essential workers) and (2) participants at higher risk of mortality if infected (e.g. adults with comorbidities and the elderly). Contrary to the open-label approach, unblinding only ‘at-risk’ participants would help maintain principles of health equity [26]. Additionally, the rate of trial drop-out would decrease, which would, in turn, allow more time to monitor and collect follow-up data from the still-blinded vaccine and placebo arms.

The implications of behavioural disinhibition when unblinding participants should be considered when adopting this approach. By choosing to unblind at-risk groups, those in the vaccine arm would be aware of their previous status, which may lead to a disinhibition of precautionary measures, thus acting as a confounding factor. To address this issue without compromising the integrity of the data, a fourth approach of blinded cross-over trials may be considered.

## Approach 4: Transitioning to a blinded stepped-wedge cross-over design

**Fig. 4:**
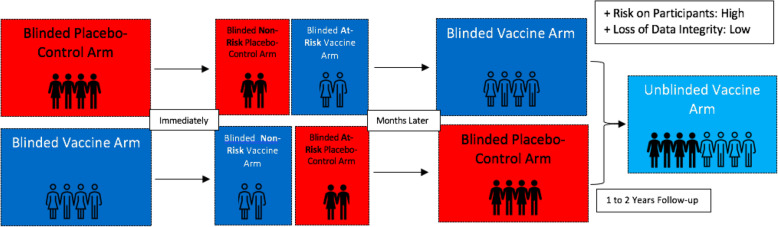
Transitioning to a blinded stepped-wedge cross-over design

In the case of large clinical vaccine trials, an elongation of the study period—during which unbiased, uncompromised data could be collected—is always of significant benefit. In a typical blinded cross-over trial, participants in the placebo arm would be provided with the vaccine, while those in the vaccine arm, would be provided with placebo (Fig. 4) [26, 27]. Neither of the two arms would be aware of their initial or current status, thus preserving the integrity of the data, while serving the interests of all participants. To further maximise data integrity, and address concerns of health equity, the trial participants may be crossed-over in phases (stepped-wedge design) based on risk-assessment (similarly to approach 3). This would prolong the duration of collected placebo-controlled data and would allow for insightful information to be gathered on duration of obtained immunity and whether there is any waning over time. Such a phased approach was adopted first in The Gambia to evaluate long-term side effects associated with hepatitis B vaccination and is accepted as a viable alternative to the typical fixed-allocation RCT design [3, 28, 29].

Nonetheless, the greater logistic and economic burden brought forth by this approach may be an obstacle to its implementation, which has already been expressed by vaccine manufacturers [20]. Additionally, several concerns would remain to be addressed: for instance, whether the cross-over would occur after phase III is finalised or as soon as logistically possible and for how long the cross-over trial would last. It would also be important to consider the type of information that will be divulged to participants without compromising the blinding and behaviour inhibition of the trial.

## Approach 5: Progressing to a blinded active-controlled stepped-wedge cross-over trial

**Fig. 5:**
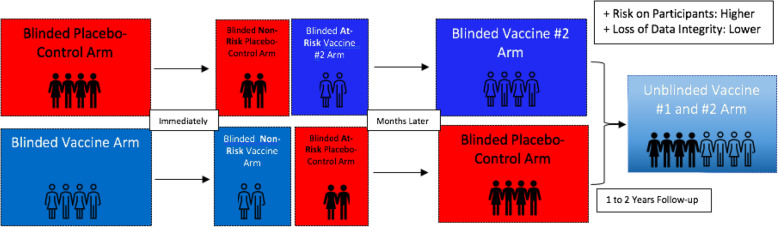
Progressing to a second blinded active-controlled stepped-wedge cross-over vaccine trial

It is important to consider that there has been no other instance in our past or modern history where a vaccine is developed and supported with good data in the midst of an ongoing pandemic. This unprecedented scenario thus invites unprecedented approaches. As such, a fifth option that we propose to address the ethical dilemma discussed is to invite the trial participants into a second cross-over trial where (1) the vaccine group is provided with placebo, and (2) the placebo group is provided with a vaccine that differs from the one used in the initial trial (Fig. 5). This vaccine however must have been proven of similar comparative efficacy in order to justify a state of equipoise. The provision of a treatment in the control group of comparative efficacy to the intervention arm, such as standard of care, is what is referred to here as an ‘active-controlled’ trial. Additionally, the crossing-over could be phased or ‘stepped’ as per approaches 3 and 4. By taking this approach, all the benefits of a blinded stepped-wedge cross-over trial would be achieved, thus maximising data integrity while ensuring that all participants receive an efficacious COVID-19 vaccine. In addition to this however, data of significant value would be collected, allowing for a comparison between vaccines and a search for maximal efficacy and minimal side effects. Likewise, if the vaccines turn out to be of similar efficacy and safety, this would reassure the public that all vaccine options are of equal benefit. This approach would be analogous to a typical randomised active-controlled trial where a therapy under question in the intervention arm is compared to a control arm receiving the standard of care (which in this case would be the vaccine arm of the initial trial and the placebo of the second). A similar approach was adopted previously in the case of leprosy vaccine trials in Vietnam, where an arm receiving the new mixtures of BCG and killed *Mycobacterium leprae* where compared to a control arm receiving the pure BCG vaccine [28].

Perhaps, it may be argued that during this COVID-19 pandemic, a search for ‘optimal’ rather than ‘satisfactory’ countermeasures is a necessity, considering the global implications and the degree of public trust in vaccination that is at stake [30]. Furthermore, although the current data from the approved vaccines suggests a significant reduction in COVID-19 disease, it remains unclear whether sterilised immunity via prevention of infection and transmission is also achieved [31]. As such, this may further justify a search for the optimal vaccine, which this approach may facilitate while circumventing the difficult logistics of an active-controlled trial from scratch. In the case of vaccine manufacturers in early stages of testing, this approach may be of even greater importance, as the argument of equipoise for new phase III placebo-controlled trials at the current time is brittle [13].

Nonetheless, it is of great importance in this approach that current vaccine trial participants are not pressured to enter a second trial and that an alternative route to immediate vaccination is made available. Additionally, side effect profiles may be more obscure in the case of vaccine mixing; as such, close auditing/monitoring by the respective committees and potential adopting/incorporation of Bayesian adaptive designs should be considered. It is also essential to emphasise and acknowledge the collaborative and well-intentioned efforts that are required by different bodies, many of which may have conflicting interests, in order for such an approach to succeed.

## Approach 6: Conducting randomised stepped-wedge community trials

Although the vitality and necessity of randomised clinical trials in public health decision making is undisputed, it is important to remember that the end goal is always to successfully transfer the knowledge gained from ideal controlled settings to the real-world field setting. In order to do so however, and to ensure the validity of RCT data in the field, a community trial should be considered. Perhaps the most pertinent application of field trials can be traced back to the poliomyelitis epidemic in the early twentieth century [32]. Despite arguably being a more dangerous and fear-instilling disease, the pressure for an efficacious and safe vaccine against poliomyelitis is not too different than the current pressure for a similar vaccine against COVID-19. Likewise, the debates regarding placebo arms and health equity carry forward to this day [32]. In community trials, data on the disease-preventing efficacy of the vaccines is collected in a wider, unaltered, public setting often with a much larger sample size, as opposed to the meticulously designed, venue- and participant-bound RCTs [28].

As of current, only 29.09% of the world population has been fully vaccinated against COVID-19 [33], with several months expected before a notable proportion of the population—especially from low-income countries—is vaccinated [34]. Due to logistic constraints, individuals who register for the vaccine would thus have to wait for a duration of time before being provided with the chance to receive it. As such, we recommend taking advantage of the current situation in designing a randomised stepped-wedge community trial via the following steps:

1. Centralising the information on individuals who have registered and/or taken the vaccine, and COVID-19 positive cases, on a national level.
2. Randomly allocating vaccination appointment from the list of registered individuals. If those at risk are to be given priority over non-risk, then randomisation should still be maintained within each risk priority cluster.
3. Continuing standard COVID-19 RT-PCR screenings, in addition to added random voluntary community screenings. Health surveys may also be provided during the random community screenings to collect data on symptoms related to vaccine side effects, regardless of vaccination status (endpoint: vaccine side-effect profile).
  - Random community screenings may also collect data on subsidiary objectives such as serological profiles, which would provide further data on immunisation at different cross-sections.
4. Monitoring the number of positive cases in the population, stratified by individuals who have received the vaccine (intervention arm), and individuals who have registered but not yet received the vaccine (control arm) (endpoint: vaccine efficacy).
  - We recommend limiting the analysis to individuals who registered for the vaccine for three main reasons:
    i. To limit potential confounding factors related to heath behaviours, attitudes and general demographics between individuals who choose to register for the vaccine and those who opt not to, thus maximising standardisation of baseline data.
    ii. To randomise the group of individuals that are allocated to receive the vaccine versus those who are not allocated to receive it, at any given time.
    iii. To create an opportunity for the collection of informed consent from those who register for the vaccine and thus participate in the community trial.
5. Comparing the incidence of disease in vaccinated individuals and registered but unvaccinated individuals in the community, in an observer-blinded analysis. This analysis would be continuous and conducted in ‘real-time’ throughout the community trial, potentially adopting a Bayesian design [35], as more individuals are vaccinated at the different phases of the stepped-wedge design.

By adopting this approach, not only is valuable public health data continued to be collected, but the ethical dilemmas are likewise addressed. Additionally, data from approach 5 regarding comparative vaccine efficacy could easily be collected and analysed through this design. Furthermore, health equity requirements would be met, due to the randomised nature and equal opportunity nature of the protocol. Additionally, note that this approach does not necessarily have to be considered after an open-label approach is adopted for the current vaccine trials, but instead may be conducted in parallel with any of the approaches listed, hence why we have left it for the end. If, however, this approach is adopted following an open-label approach, the concerns regarding irreplaceable loss of data integrity with the loss of placebo arms would remain.

## Conclusion

**Fig. 6:**
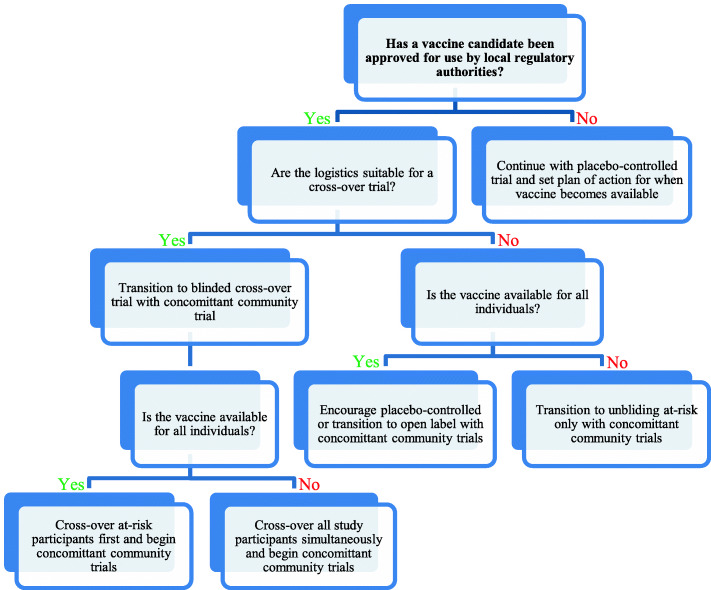
Algorithm for decision making in advanced stages of vaccine trials

Ethical dilemmas in clinical research continue to pose a delicate challenge. The actions taken by the medical community in line with the underlying foundational desire to do the greatest good for society and what is in the best interest of participants is what differentiates modern medicine from past eras of unaccountability. In the case of COVID-19, if possible, new vaccine trials entering phase III should compare the efficacy of their vaccine to an arm receiving another vaccine approved for use by local regulatory bodies. If none exist, then a placebo-controlled arm may be justified in pursuit of optimal vaccine characteristics. As for long-term trial continuation of approved vaccines, a risk-prioritised stepped-wedge transition to a blinded crossover trial may be the best option to ensure all at risk participants are given an effective vaccine without compromising blinded status, thus maximising the scientific gains with the participants’ best interest in mind (Fig. 6). Where logistically suitable, an active-controlled cross-over vaccine trial should also be considered. Finally, community trials (preferable adopting a randomised stepped-wedge design) should be conducted as soon as feasibly possible. The role of ethical review boards in the approval and auditing of vaccine trials is of imminent importance in the current context and should be emphasised; final consensus on the most ethically appropriate and scientifically acceptable designs and their alternatives should be rigorously evaluated, taking into consideration the unique local contexts, and in partnership with a diverse group of experts and stakeholders [22]. It is important to remember that the emergent nature of the COVID-19 situation does not justify a compromise on core ethical values. In fact, the discourse surrounding this topic and the decisions made will remain a potent case study and a continuously referenced example for all such future scenarios.

## Data Availability

Not applicable.
